# Disease Site-Specific Guidelines for Curative Radiation Treatment During ‘Limited Surgery’ and ‘Hospital Avoidance’: A Radiation Oncology Perspective From the Epicenter of COVID-19 Pandemic

**DOI:** 10.7759/cureus.8190

**Published:** 2020-05-18

**Authors:** Bhupesh Parashar, William C Chen, Joseph M Herman, Louis Potters

**Affiliations:** 1 Radiation Oncology, Zucker School of Medicine at Hofstra/Northwell, Lake Success, USA; 2 Radiation Medicine, Zucker School of Medicine at Hofstra/Northwell, Lake Success, USA

**Keywords:** coronavirus, radiation, oncology, curative, pandemic, guidelines, covid-19

## Abstract

The COVID-19 pandemic has resulted in an unprecedented situation where the standard of care (SOC) management for cancers has been altered significantly. Patients with potentially curable cancers are at risk of not receiving timely SOC multidisciplinary treatments, such as surgery, chemotherapy, radiation therapy, or combination treatments. Hospital resources are in such high demand for COVID-19 patients that procedures, such as surgery, dentistry, interventional radiology, and other ancillary services, are not available for cancer patients. Our tertiary care center is considered the center of the epicenter in the USA. As a result, all non-emergent surgeries have been suspended in order to provide hospital beds and other resources for COVID-19 patients.

Additionally, ambulatory efforts to avoid treatment-related morbidity are critical for keeping patients out of emergency departments and hospitals. In this review article, we discuss evidence-based radiation therapy approaches for curable cancer patients during the COVID-19 pandemic. We focus on three scenarios of cancer care: 1) radiation therapy as an alternative to surgery when immediate surgery is not possible, 2) radiation therapy as a ‘bridge’ to surgery, and 3) radiation options definitively or postoperatively, given the risk of hospitalization with high-dose chemotherapy.

## Introduction and background

The main challenge faced during the coronavirus disease 2019 (COVID-19) pandemic is the need to maintain access to proper cancer treatment. In order to achieve access, the staff providing those services must be safe, healthy, and available. The pandemic has resulted in an unforeseen and unprecedented situation where major hospitals and academic centers are overwhelmed with COVID-19 patients, with limited access to standard of care (SOC) cancer treatments. For example, most operations are being postponed in order to provide hospital capacity for COVID-19 patients and also to reduce the risk of COVID-19 infection in patients that undergo surgery for non-emergent clinical situations. Amid this social and medical upheaval, there is concern and confusion regarding the best way to manage the treatment of potentially curable cancer patients. Recommendations regarding palliative treatment for incurable cancer cases are published elsewhere [1-2]. 

In this review, we discuss radiation therapy (RT) options for cancer patients in three settings: 1) RT as an alternative to surgery when immediate surgery is not possible, 2) RT as a 'bridge' to surgery and 3), radiation options definitively or postoperatively, given the risk of hospitalization with high-dose chemotherapy.

Proposed principles to guide the approach to cancer care 

The proposed guidelines are evidence-based and are being successfully used in our major and the largest tertiary care academic health center in New York, the center of the epicenter. 

In general, cancer patients who may be at an increased risk of having more complications from COVID-19 include those with uncontrolled diabetes, chronic lung infections, and patients currently on cancer treatments.

In order to minimize the risk of exposure to COVID-19 and maintain high-quality patient care with optimal disease outcomes, we have used the following principles to guide our institutional approach to cancer care:

1. When available, test cancer patients and staff for coronavirus infection and exposure 

2. Follow or create safety protocols to prevent infections for patients and staff

3. Multidisciplinary discussion for each patient case (virtual or in-person with social distancing) 

4. Short-course hypofractionated RT is preferred when feasible

5. Conservative (smaller) RT fields are preferred, if possible, to minimize RT-induced lymphopenia and reduce mucosal toxicity

6. Consider enrollment into clinical trials to collect data and information on this cohort of patients. Consider enrollment onto clinical trials if infected with the coronavirus.

Also, the following general recommendations should be considered while treating cancer patients with radiation during COVID-19:

1. Aggressive and preemptive management of side effects

Includes early interventions, such as skin creams, anti-diarrheal, cough suppressant, anti-inflammatory, and nutritional supplements

2. Low threshold for hydration, pain management

3. Enhanced communication (consider virtual) with multidisciplinary teams (medical oncology, surgery, nutrition, occupational and physical therapy [OT/PT], social work, and wound care)

## Review

Impact of radiation therapy on the immune system

Radiation is considered immunosuppressive due to the sensitivity of lymphoid cells to radiation-induced cell death. However, given the focused nature of current external beam radiation therapy (EBRT) techniques, the quantitative effect of RT on the immune system is limited. In contrast, systemic treatments, such as chemotherapy, monoclonal antibodies, small molecule inhibitors, and immunotherapy, can result in substantial reductions in host immunity due to systemic effects on bone marrow and circulating blood cells [3]. Conformal EBRT results in limited exposure of healthy tissues to radiation for most sites. Therefore, the clinically significant immune suppression caused by conformal radiation therapy, e.g., three-dimensional conformal radiation therapy (3DCRT), intensity-modulated radiation therapy (IMRT), and stereotactic body radiation therapy (SBRT), is limited when compared to chemotherapy, as commonly seen in clinical settings (4). In cases where patients are treated with large EBRT fields that include a significant amount of bone marrow, e.g., palliative RT to spine or pelvis, peripheral blood counts can be compromised [4]. 

Recommendations for cancer care during COVID-19 pandemic: curative treatments

Curative treatments should be prioritized based on the tumor site, histology, performance status, and stage. In general, the American Society of Clinical Oncology (ASCO) and the American Society of Therapeutic Radiation Oncology (ASTRO) guidelines suggest that specific non-emergent treatments for early-stage prostate and breast cancer should be delayed [5-8]. In other cases, there are few recommendations related to the current COVID situation. Therefore, decisions regarding management should be based on a multidisciplinary discussion with the entire team and intended for the current COVID-19 crisis (or future similar crises) when surgical treatments and chemotherapy are not readily available or are not preferred based on the patient's risk profile. The recommendations we propose are not always considered the SOC during ‘non-crisis’ settings but are meant to maintain high-quality care and optimal disease outcomes for curative cases. 

The recommendations below assume surgery is not available or not recommended based on the patient’s risk profile, and full-dose chemotherapy may be risky given systemic side effects and risk of patient hospitalization. In all situations where concurrent chemotherapy is a standard approach, it should be continued if the multidisciplinary team agrees that the risk of toxicity is low and the toxicities can be easily managed in an ambulatory setting without exposing the patients to unnecessary risks of hospitalization. If the risk of hospitalization is high, a different approach should be used: 1) sequential chemotherapy and radiation, 2) reduction of chemotherapy dose, 3) use of a less toxic concurrent systemic or targeted therapy, or 4) RT alone with an option of altered fractionation to compensate for lack of chemotherapy. 

Head and Neck Cancers

Head-and-neck cancers (HNC), most commonly squamous cell carcinoma (SCC), are usually considered high-risk for local progression and need to be managed sooner than later. All decisions should be based on a multidisciplinary management approach. In patients who are considered candidates for RT alone, treatment may be started with a focus on hydration, minimizing the risk of hospitalization by excellent nutritional support and social support. Pre-treatment dental evaluation should be performed if possible but could be a challenge during the pandemic, given the lack of resources and risk of infections to patients and health care workers. Patients should be made aware of the challenges of not performing proper dental evaluation before RT and the associated risk of osteonecrosis and worsening dentition. Dental guards can be used to reduce electron scatter and reduce toxicity. 

Older Patients with HNC

Older patients (> 70 years old) who are unable to undergo surgery or concomitant chemoradiation for the advanced disease may be treated with hypofractionated RT or SBRT, 35 - 44 Gy in five fractions delivered every other day. However, other fractionations are also reported to show acceptable results [9-11]. RT plans should be conformal, keeping the mandible and dental dose as low as reasonably possible. In these situations, the gross tumor should be covered as a part of the primary planning target volume (PTV) and the elective nodal region avoided to minimize the risk of severe toxicity that may result in hospitalization. Although RT should be completed in the shortest possible time, older patients that develop toxicities may need to be given a break from treatment rather than continuing and putting them at risk of hospitalization in a high-risk COVID-19 patient-filled hospital.

Oropharyngeal Cancer

For human papillomavirus (HPV)-associated oropharyngeal cancer, RT alone is an option for early-stage disease (70 Gy in 2 Gy/fraction). For HPV-negative disease, concomitant chemo-RT should be standard of care, with modified chemotherapy/targeted dose regimens. If chemotherapy is not possible, RT alone (using altered fractionation, dose-escalation, or SBRT) may be appropriate [12]. 

Laryngeal Cancer

For laryngeal cancers (supraglottic, subglottic) and hypopharyngeal cancers, EBRT alone may be utilized (70 Gy in 2 Gy per fraction). For glottic cancers, RT to 63 Gy in 28 fractions is the standard and should be followed [12]. 

Nasopharyngeal Cancer

For nasopharyngeal cancers, RT with chemotherapy may be preferred given survival benefit seen in multiple studies compared to RT alone, although RT alone should be used if systemic therapy is risky and cannot be used (70 Gy in 2 Gy/fraction) [12]. 

Salivary Gland Cancer

For parotid cancers, primary surgery is the preferred treatment. Therefore, delaying treatment for a few weeks until surgery can be performed may be a reasonable approach. However, if the patient is unable to wait or the tumor is progressing, EBRT (70 Gy in 2 Gy/fraction) or SBRT (35 - 44 Gy in five fractions) may be utilized [12]. A similar rationale can be applied to other salivary gland cancers. 

Oral Cavity Cancer

Oral cavity cancers are primarily managed with surgery, and efforts should be made to wait if surgery cannot be performed immediately. Other options include induction chemotherapy, preoperative radiation, or definitive EBRT/SBRT. Preoperative RT is a good option if surgery is being considered within four to six weeks after completion of RT (50 Gy in 2 Gy/fraction) [11]. This rationale may be valid for the paranasal sinus tumor as well.

Postoperative Head and Neck Cancers

For high-risk HNC post-resection, adjuvant RT alone may be preferred to chemoradiation to limit toxicity (54 - 66 Gy in 1.8 - 2 Gy/fraction). For patients with multiple nodes, extra-capsular extension (ECE), and other high-risk features where adding chemotherapy is the standard [12], modified chemotherapy doses or less toxic regimens may be used in consultation with the medical oncology team. If adding chemotherapy puts the patient at risk of potential hospitalization, RT alone may be the preferred approach.

ASTRO/European Society of Therapeutic Radiation Oncology (ESTRO) consensus practice recommendations for head and neck cancers during the COVID-19 pandemic were recently published [5-6].

Thoracic Cancers

Small cell lung cancers are usually treated with a combination of chemotherapy and radiation to the thorax for limited-stage disease, followed by prophylactic cranial irradiation (PCI). For extensive-stage disease, chemotherapy followed by thoracic RT and PCI may be offered for those responding to chemotherapy [12]. RT alone to the chest should be considered if chemotherapy is challenging. PCI may be given for both limited-stage and extensive-stage disease.

RT dose to the thorax may be 60 - 70 Gy in 1.8 - 2 Gy once a day fraction for limited-stage cancer and 30 Gy in 10 fractions to the thorax for the extensive-stage disease. The PCI dose is 25 Gy in 10 fractions [12]. 

For patients with peripheral stage I/IIA non-small cell lung cancer, SBRT may be an appropriate option. Given a biopsy of the lesions may be challenging, SBRT without pathologic confirmation may be considered after a multidisciplinary discussion if a biopsy is not available or if the patient is at a high risk of complications from a biopsy. The National Comprehensive Cancer Network (NCCN) recommended SBRT dose options are listed below (12). Options utilizing fewer fractions may be preferable to limit patient visits to the department (Table 1). 

**Table 1:** NCCN Recommendation for SBRT Doses for Early-Stage Lung Cancer [12] NCCN: National Comprehensive Cancer Network; SBRT: stereotactic body radiation therapy

Total dose (Gy)	Fraction #	Examples
25 - 34	1	Peripheral, < 2 cm, preferably away from the chest wall
45 - 60	3	Peripheral, > 1 cm from the chest wall
48 - 50	4	Central or peripheral, < 4 - 5 cm, < 1 cm from the chest wall
50 - 55	5	Central or peripheral tumors, < 1 cm from the chest wall
60 - 70	8 - 10	Central tumors

For patients with stage IIB/III non-small cell lung carcinoma (NSCLC), sequential radiation and chemotherapy may be considered. The standard dose is 60 Gy in 2 Gy per fraction. Recommended RT doses for definitive treatment, preoperative treatment, and postoperative RT are listed in Table 2 [12]. Postoperative RT for N2 disease (the presence of ipsilateral mediastinal nodal metastases) may be used for patients with multilevel nodal involvement with ECE or other high-risk features (Table 2). There are recently published consensus guidelines for thoracic cancers [7].

**Table 2:** Recommended Doses for Definitive RT and Postoperative RT for Non-Small Cell Lung Cancer [12] RT: radiation therapy

Type of treatment	Total dose	Fractional dose
Definitive with or without chemotherapy	60 - 70 Gy	2 Gy
Preoperative	45 - 54 Gy	1.8 - 2 Gy
Postoperative RT, extracapsular extension or positive margins, gross residual disease	50 - 54 Gy, 54 - 60 Gy, or 60 - 70 Gy	1.8 - 2 Gy, 1.8 - 2 Gy, or 2 Gy

Thymic Tumors

Surgical resection is the SOC, and radiation is offered postoperatively for high-risk categories. Postoperative RT may be omitted for low-risk thymomas (e.g., stage I thymoma). For higher-risk thymomas, preoperative radiation may be used if surgical resection is not available at this time with an intention to operate within a few weeks of completing RT. Chemotherapy may be added preoperatively or postoperatively for high-risk clinical presentations [13-14]. 

As per the NCCN, the adjuvant RT dose is 45 to 50 Gy for negative margins, 54 Gy for microscopically-positive margins, and 60 - 70 Gy for gross residual disease in conventional fractionation (1.8 - 2.0 Gy/fraction) [12]. For medically inoperable patients, a dose of 60 - 70 Gy in 1.8 - 2 Gy/fraction is recommended. For patients unable to undergo surgery due to constrained resources from the pandemic, a preoperative RT/chemoradiation therapy (CRT) approach can be used with 40 - 45 Gy in conventional fractionation [13-14].

Esophagus Cancer

If surgery or concurrent chemoradiation is a challenge, RT alone or induction chemotherapy may be an option [12]. Preoperative RT should be considered if surgery may become available in a few weeks once the COVID-related risks diminish. Limiting RT fields to the gross tumor, plus margins may reduce the risk of treatment-related toxicity.

Recommended RT doses [12]:

Preoperative RT: 41.4 - 50.4 Gy in 1.8 Gy/fraction

Definitive RT: 50 - 50.4 Gy in 1.8 - 2 Gy/fraction. If chemotherapy is not planned, EBRT alone to a dose of 60 - 64 Gy in 1.8 - 2 Gy/fraction can be used. 

Postoperative RT: 45 - 50.4 Gy in 1.8 - 2 Gy/fraction

Gastric Cancer

Gastric cancer is primarily a surgically managed disease. Perioperative options include neoadjuvant chemotherapy, neoadjuvant chemoradiation, adjuvant chemotherapy, and adjuvant chemoradiation. During the pandemic, if surgery and chemotherapy are constrained, the role of RT becomes even more critical.

If surgery is not available, preoperative radiation can be utilized to delay surgical intervention. There are several studies showing a good pathological response to preoperative RT [15-17]. This strategy may help reduce tumor burden and also serve as a bridge until surgery and chemotherapy become available. A Chinese study evaluated preoperative RT to surgery alone and showed significant local control and survival benefit to adding preoperative RT [15]. The RT dose in the study was 40 Gy in conventional fractionation to the cardia, lower segment of the esophagus, fundus, lesser curvature, and hepatogastric ligament. Additional studies have shown the benefit of preoperative radiation with chemotherapy [16-17]. 

Preoperative radiation dose: 39 - 42 Gy in 1.8 - 2 Gy per fraction

Postoperative RT: 45 - 50.4 Gy in 1.8 - 2 Gy per fraction [12]

Pancreatic Cancer

Standard fractionated chemoradiation has been shown to prevent local recurrence in the adjuvant setting and decrease local progression in the locally advanced setting [18-19]. In patients with borderline resectable pancreas cancer, neoadjuvant chemotherapy and radiation have resulted in margin-negative resection rates and overall survival that is comparable to those patients who present with a resectable disease [20]. While studies have shown similar outcomes when RT is given concurrently with gemcitabine or capecitabine, capecitabine results in a lower risk of neutropenia and immunosuppression and, therefore, should be preferred during the current pandemic [21]. 

Multiple single and multi-institutional studies have shown that SBRT given over three to five days in 8 - 12 and 5 - 8 Gy fractions, respectively, results in survival outcomes that are similar to chemoradiation with less toxicity [22]. If fiducial placement is challenging, pre-treatment images, such as magnetic resonance imaging (MRI), computed tomography (CT) scan, or positron emission tomography-computed tomography (PET-CT), should be fused with a treatment planning scan. If possible, it is preferable to use shorter RT treatment delivery times by using manual breath-hold (with coaching) and abdominal compression as opposed to cone-beam computed tomography (CBCT) or MRI as it can take longer to deliver the therapy. Specifically, if there is no duodenal or stomach invasion, doses less than 8 Gy x 5 or 10 Gy x 3 can be given safely without fiducials or specialized imaging (MRI). However, larger SBRT doses should not be delivered if there is bowel/stomach invasion, with or without either fiducial, image-guided radiation therapy (IGRT), gating, and MR-guided RT [23].

According to the NCCN, for resectable preoperative chemoradiation, the following RT doses have been reported: 36 Gy in 2.4 Gy fractions to 45 - 54 Gy in 1.8 - 2.0 Gy fractions [12]. Surgery may be performed four to eight weeks after RT. For resected pancreatic adenocarcinoma, the RT dose generally consists of 45 - 46 Gy in 1.8 - 2.0 Gy fractions to the tumor bed, surgical anastomoses (hepaticojejunostomy and gastrojejunostomy may be omitted if clinically appropriate), and adjacent lymph node basins, potentially followed by an additional 5 - 9 Gy to the tumor bed, and anastomoses, if clinically appropriate [12]. In rare occurrences, SBRT could be used in the adjuvant setting if there is a gross residual disease (R2 resection) that can be targeted based on anatomy or surgically placed clips and bowel is not in the field. For unresected pancreatic adenocarcinomas, a short course of SBRT is appropriate. NCCN cites SBRT doses of three fractions (total dose: 30 - 45 Gy) or five fractions (total dose: 25 - 45 Gy) [12, 23]. 

Colon Cancer

If chemotherapy and surgery are not possible or best avoided given the hospital's resources, RT may be used as a regimen for local control, although it is certainly not the standard [24]. If resection has already been performed, RT may be directed to the tumor bed for high-risk diseases, such as T4 disease due to penetration to a fixed structure. For postoperative RT, 45 - 50.4 Gy can be delivered in 1.8 - 2 Gy per fraction. Preoperative RT may be utilized as a bridge to the time surgery may be performed with concomitant chemotherapy, if possible (or without chemotherapy if the combination is risky), in 45 - 50.4 Gy in 1.8 - 2 Gy/fraction [25-26].

If surgery is not being planned (given the pandemic) and abdominal malignancy is progressing, RT using either IMRT or 3D may be utilized, making sure that abdominal organs at risk (OAR) constraints are met. The use of SBRT in primary colon cancer has limited evidence. However, there is prospective evidence that has evaluated SBRT in abdominal metastasis [27]. 

Gynecological Cancers

Pelvic RT and vaginal brachytherapy are usually well-tolerated and can be used in the majority of patients without a need for hospitalization. 

Endometrial cancers cancer be treated with EBRT and vaginal brachytherapy [12]. As per the NCCN recommendations, ‘external-beam doses for a microscopic disease should be 45 - 50 Gy in conventional fractionation (1.8 - 2 Gy/fraction). Postoperatively, if there is a gross residual disease and the area(s) can be sufficiently localized, a boost can be added to a total dose of 60 - 70 Gy, respecting normal tissue sensitivity. For neoadjuvant radiation, doses of 45 - 50 Gy are typically used. One could consider adding one to two high-dose-rate (HDR) insertions to a total dose of 75 - 80 Gy low-dose-rate (LDR) equivalent, to minimize the risk of positive or close margins at hysterectomy.’ 

As per the NCCN [12], for patients receiving postoperative HDR vaginal brachytherapy alone, regimens include 6 Gy x 5 fractions prescribed to the vaginal surface, or 7 Gy x 3 fractions or 5.5 Gy x 4 fractions prescribed to 5 mm below the vaginal surface. The use of smaller fraction sizes may be considered to potentially further limit toxicity in selected cases. 

Doses of 4 - 6 Gy x 2 - 3 fractions are prescribed to the vaginal mucosa if HDR is used as a boost to external beam RT.

For medically inoperable uterine cancer or patients where surgery cannot be performed, an EQD2 (equivalent dose in 2 Gy fraction) D90 of at least 48 Gy should be delivered to the uterus, cervix, and upper 1 - 2 cm of the vagina if brachytherapy alone is used and should be increased to 65 Gy for the combination of EBRT and brachytherapy. If an MRI is used as part of planning, the target dose for the gross tumor volume (GTV) would be an EQD2 of ≥ 80 Gy [12].

Cervical cancer can be treated with reduced-dose chemotherapy, plus EBRT or EBRT alone. As per the NCCN, patients with an intact cervix are typically treated with definitive EBRT to a dose of approximately 45 Gy (40 - 50 Gy in conventional fractionation) followed by a brachytherapy boost. However, since brachytherapy for cervix cancer may be difficult, given the utilization of resources for COVID-19 infections, an IMRT or SBRT boost may be applied. SBRT does not usually replace brachytherapy. However, it may be an appropriate option if brachytherapy cannot be performed. The SBRT dose may be determined by published retrospective and phase I and II data [28-29].

Rectal Cancer

For patients that are scheduled to undergo surgical resection and health system resources permit, preoperative short-course RT treatment may be preferred. The NCCN recommends a short course of pelvic RT for T3 and M1 (if symptomatic or controlled systemic disease) rectal cancer. 

Short-course radiation therapy (25 Gy in five fractions) with surgery within one week of completion of therapy or delayed six to eight weeks can also be considered for patients with stage T3 rectal cancer [12].

Another option for preoperative radiation is 45 Gy to the pelvis, followed by 5.4 Gy in three fractions to the tumor with a 2 cm margin. IGRT is preferred in this setting to limit the dose to the adjacent bowel. 

Conventional fractionation is the standard for postoperative rectal cancer patients. A dose of 45 Gy to the tumor bed and a boost of 5.4 - 9.0 Gy in three to five fractions are used for postoperative radiation.

For unresectable cancer, RT alone may be an option. Dose as per the NCCN is > 54 Gy in conventional fractionation. Chemotherapy or systemic treatment may be added based on the risk of toxicity.

Anal Cancer

Standard treatment of anal cancer is concomitant chemoradiation. However, RT alone may be an acceptable option if adding chemotherapy may result in a higher risk of patients being hospitalized [30]. 

RT dose options as per the NCCN include the “shrinking field technique” with low-risk elective nodal PTV volume prescribed to 30.6 Gy in 1.8 Gy daily fractions [12]. The high-risk elective nodal PTV is sequentially prescribed an additional 14.4 Gy in 1.8 Gy/fraction for a total prescribed dose of 45 Gy. Finally, for T1-2 lesions with residual disease after 45 Gy, T3-4 lesions, or N1 lesions, an additional 5.4 - 14.4 Gy in 1.8 - 2 Gy per fraction is sequentially prescribed to a total dose 50.4 - 59.4 Gy. Protons may be an option for both anal and rectal cancer in institutions that have this modality (Table 3) [31].

**Table 3:** Radiation Therapy Oncology Group (RTOG) 0529 Recommendations [12] fx: fraction; PTV: planning target volume; TNM: Tumor Node Metastasis

TNM stage	Primary tumor PTV dose	Nodal PTV dose
T2N0	50.4 Gy (28 fx)	42 Gy (28 fx)
T3-4N0	54 Gy (30 fx)	45 Gy (30 fx)
Any T, N+ (< 3 cm)	54 Gy (30 fx)	50.4 Gy (30 fx)
Any T, N+ (> 3 cm)	54 Gy (30 fx)	54 Gy (30 fx)

For patients that are elderly, some additional RT dose options have been published based on retrospective data, e.g., 30 Gy in 15 fractions with concurrent 5-fluorouracil (5FU) [32], although this study included only a small number of patients and should not be considered a standard option.

Skin Cancer

RT for primary and resected skin cancers can be delayed. However, if RT is indicated, use short courses and limit radiation to the mucosa. Elective nodal radiation should be avoided unless strongly indicated. When using a large fractional dose, alternate day or less frequent RT treatment can be utilized. If surgery is not possible soon, primary RT can be utilized.

Unresected Squamous Cell/Basal Cell Skin Cancers

For primary tumors less than 2 cm, one can use 30 Gy in five fractions over two weeks [12].

For a primary tumor > 2 cm, 45 - 55 Gy delivered over three to four weeks can be used [12]

10.2 Gy per fraction x 3 (weekly) [33]

Resected Squamous Cell/Basal Cell Skin Cancers

50 Gy in four weeks (2.5 Gy/fraction) [12]

44 Gy in 10 fractions four days a week [34]

Melanoma [12]

Definitive cases: 35 Gy in five fractions over one week for < 3 cm^2^

Postoperative: 30 Gy in five fractions twice a week or every other day.

Soft-Tissue Sarcomas

Soft tissue sarcomas can be treated with preoperative RT if surgery cannot be performed, given the coronavirus pandemic. If surgery has already been performed, postoperative RT can be delivered in high-risk pathology. Brachytherapy may be difficult at this time and may need to be substituted by EBRT. Most of the RT recommendations are based on extremity sarcomas, although the rationale can be applied to non-extremity sites.

The NCCN recommends the following RT doses that can be utilized during the COVID-19 pandemic [12]:

Preoperative RT: 50 Gy EBRT in conventional fractionation. Following preoperative 50 Gy EBRT and surgery, for positive margins, consider observation or RT boost. If using RT boost, consider EBRT: 16 - 18 Gy for microscopic residual disease or 20 - 26 Gy for gross residual disease. 

Postoperative RT doses are EBRT (50 Gy) + EBRT boost [12]: Negative margins: 10 - 16 Gy Microscopically positive margins: 16 - 18 Gy

Gross residual disease: 20 - 26 Gy

Besides, there are reports of using SBRT as a preoperative regimen (e.g., 35 Gy in five fractions) for sarcomas [35-36]. 

Prostate Cancer

During this COVID-19 pandemic, hypofractionated regimens may be preferred to reduce patient visits and limit interactions. Intermediate and high-risk patients can have combined androgen deprivation therapy (Table 4).

**Table 4:** The NCCN Recommends the Following RT Doses for Low, Intermediate, and High-Risk Prostate Cancer [12] fx: fraction; NCCN: National Comprehensive Cancer Network; RT: radiation therapy

Regimen	Preferred dose/fractionation	Very low and low risk	Favorable intermediate risk	Unfavorable intermediate	High and very high-risk	Regional N1	Low volume M1
Moderate hypofractionation	3 Gy x 20 fx, 2.7 Gy x 26 fx, or 2.5 Gy x 28 fx	yes	yes	yes	yes	yes	2.75 x 20 fx
Conventional fractionation	1.8 - 2 Gy x 37 - 45 fx	yes	yes	yes	yes	yes	
Ultra-hypofractionation	7.25 - 8 Gy x 5 fx or 6.1 Gy x 5 fx	yes	yes	yes	yes	yes	6 Gy x 6 fx

At our institution, we commonly use 42.5 Gy in five fractions for appropriately selected patients [37]. 

Usually, SpaceOAR® hydrogel (Augmenix, Inc., Bedford, MA) and fiducial (or Calypso®, Varian Medical Systems, Palo Alto, CA) markers are placed before SBRT. However, given the pandemic and lack of resources, these placements can be done under local anesthesia or skipped. If omitted, daily CBCT or onboard imaging is recommended during RT. 

Kidney Cancer

Surgical resection is the mainstay of treatment for this cancer. For patients that cannot undergo surgical resection, given the pandemic, SBRT may be used for unresected renal cancer. The studies utilizing SBRT for kidney cancer are shown in Table 5.

**Table 5:** Studies Using SBRT for Kidney Cancer CR: complete response; DC: distant control; DLT: dose-limiting toxicity; EGFR: epidermal growth factor receptor; fx: fraction; G1: grade 1; G2: grade 2; LC: local control; OS: overall survival; PD: progressive disease; PR: partial response; pts: patients; SD: stable disease; Y: year

Authors	Study type	# patients	Stage	Dose	outcomes	toxicity
Siva et al. [38]	Prospective	37	Unresectable cancer	26 Gy/1 fx or 14 Gy/3 fx	LC 100% at 2 y, DC 89% at 2 y, OS 92% at 2 y	G1 and G2 toxicities
Staehler et al. [39]	Prospective, case-control	40	Unresectable	25 Gy/1 fx	PR/CR 38 pts, CR 19 pts	G1 toxicities
Ponsky et al. [40]	Prospective phase 1	19	Poor surgical candidates	24 - 48 Gy/4 fx	SD 12 pts, PR 3 pts at 13.7 months	Grade 2-4 toxicity, no DLT
McBride et al. [41]	Prospective phase 1	15	Medically inoperable	21 - 48 Gy/3 fx	SD 11 pts, PR 2 pts, CR 1 pt, PD 1 pt	Decline in EGFR and differential renal function
Svedman et al. [42]	Phase 2	30	Medically inoperable	32 - 45 Gy in 3 - 4 fx	CR 21%, PR/SD 58% at 52 months OS 32 months	96% G1-2 side effects

Bladder Cancer

Due to the COVID-19 pandemic, if surgery and chemotherapy are not feasible, hypofractionated short course radiation schedules can be utilized for unresected bladder cancers.

Amestoy et al. found evidence that the use of hypofractionated RT showed reasonable outcomes [43]. The NCCN recommends conventional fractionation treating the whole bladder with or without pelvic nodes to 39.6 - 50.4 Gy using conventional or accelerated hyperfractionation, followed by a boost to either the whole or partial bladder between 60 - 66 Gy. Reasonable alternatives to conventional fractionation include hypofractionated treatment of the whole bladder to 55 Gy in 20 fractions or using simultaneous integrated boosts to sites of gross disease [12]. 

High-Grade Glioma

The standard of care of primary high-grade brain gliomas is surgical resection followed by RT with concurrent temozolomide. Given the ongoing pandemic, if resection is not possible, a multidisciplinary discussion should guide each patient’s treatment. 

For resected cases, standard RT dose, based on the patient selection using a molecular profile, such as IDH (isocitrate dehydrogenase) status, is 60 Gy in 1.8 - 2 Gy/fractions [12]. 

If conventional fractionation is difficult to deliver, a decision needs to be made about an alternative regimen. The NCCN recommends the following RT doses: A slightly lower dose, such as 54 - 55.8 Gy in 1.8 Gy or 57 Gy in 1.9 Gy fractions, can be applied when the tumor volume is substantial (gliomatosis), there is brainstem/spinal cord involvement, or for grade III astrocytoma. If a boost volume is used, the initial phase of the RT plan will receive 46 Gy in 2 Gy fractions or 45 - 50.4 Gy in 1.8 Gy fractions. The boost plan will typically then receive 14 Gy in 2 Gy fractions or 9 - 14.4 Gy in 1.8 Gy fractions.

In poorly performing patients or elderly patients, a hypofractionated accelerated course should be considered with the goal of completing the treatment in two to three weeks. Typical fractionation schedules are 34 Gy/10 fractions or 40.05 Gy/15 fractions. Alternatively, a shorter fractionation schedule of 25 Gy/5 fractions may be considered for elderly and frail patients with smaller tumors for whom a longer course of treatment would not be tolerable. These hypofractionated regimens should be considered if conventional fractionation is difficult or not possible [12]. 

Several hypofractionated regimens are published for unresected or recurrent meningioma. Regimens include 36 Gy/9 fractions, 24 Gy/3 fractions, 25 Gy/5 fractions, 21 Gy/3 fractions, and 30 Gy/5 fractions [44]. These regimens are not validated in phase III randomized trials but are published in one or more studies.

Breast Cancer

Recommendations regarding RT for breast cancer during this pandemic have recently been published by an international collaborative group [45]. Recommendations include the omission of RT in older patients with early-stage, low-risk breast cancer, using hypofractionated regimens (e.g., 28 - 30 Gy in once weekly fractions over five weeks or 26 Gy in five daily fractions over one week as per the FAST and FAST Forward trials, respectively), the omission of a boost in select patients, omission of nodal radiation in select patients, and using moderate hypofractionation for RT to the chest wall (40 Gy in 15 fractions). One of the risks of adding RT postoperatively is radiation pneumonitis that may mimic COVID-19-induced pneumonia and this risk should be considered while planning RT. 

Radiation delivery

Regarding radiation delivery, protocols need to be in place to streamline therapy staff. Social distancing using technology, remote meetings, remote dosimetry, and personal protective equipment (PPE) are all techniques to enhance the safety of radiation personal [5]. 

Stress reduction and burnout

This unexpected pandemic has caused intense and widespread panic and anxiety worldwide. Wellness sessions and techniques should be used to reduce stress among radiation staff and patients, with scheduled instructional sessions held in-person or online. Staff should be made aware of available mental support resources within the institution. Patients and staff alike need ready access to mental health services. 

Imaging

Before the start of RT, adequate imaging should be obtained for primary staging. It is preferable to obtain imaging at a facility that offers online image review to minimize handling of CDs (compact discs) and other physical documents. During RT, one way to reduce exposure to staff and minimize the time of the patient in the department is to reduce the frequency of CBCT imaging from daily to weekly CBCT or weekly orthogonal films especially in circumstances where motion is minimal (brain lesions). 

Resident safety and education

Resident education is crucial, and all efforts should be made to minimize disruption in their training. With the availability of online audio and video conferencing tools, didactic lectures should continue. Online education makes it easier for many attendings and guest speakers to provide didactic instruction from local offices, thereby saving travel time and transportation/lodging expenses. 

Peer review

Peer review of plans and contours before the start of radiation can be done by remote login from each workstation and does result in 'safe distance' meetings. Institutions and departments have to make sure that the quality of meetings is good, with minimum interruptions due to lack of audiovisual quality. Otherwise, it defeats the purpose of the meeting and may necessitate a more risky in-person meeting.

Clinic flow and efficiencies

Telemedicine has been used and should be increasingly used to reduce patient exposure. The patient-physician experience is enhanced if there is real-time two-way video and audio communication [5, 46]. Even without the video component, a telephonic consult, follow-up, or weekly evaluation using telemedicine is reasonable, given the easy availability of electronic records. In places where electronic medical records may not be available, it may be necessary to gather information over the phone from referring physicians and to access radiology and pathology records through other means. 

## Conclusions

The current COVID-19 pandemic represents an unprecedented era in healthcare with potential impacts upon cancer patients. The overriding goal of maintaining access to radiation therapy during the crisis is critical, given the opportunity for radiation therapy to be curative in many situations. The lack of access to surgery and some systemic therapies impacts the ability to provide current standards of care. Nonetheless, in this review, we summarize treatment opportunities and supporting evidence that may be considered when access is limited. We must continue to care for patients during this time and reduce the potential stage migration and associated morbidity of delayed treatments. These treatment options are not to be considered a replacement of standard cancer care, but as potential alternatives when other resources are constrained. 
